# Real-world data on avelumab in first-line maintenance therapy for advanced or metastatic urothelial carcinoma: the SOGUG-AVELUMAB RWD study

**DOI:** 10.1007/s12094-025-03978-y

**Published:** 2025-07-09

**Authors:** Ovidio Fernández, Aurea Molina, Urbano Anido-Herranz, Carlos Alvarez, Regina Gironés, Pablo Gajate, Rocío García Domínguez, Silverio Ros, Javier Puente, Sara Blasco, Martín Oré-Arce, Esther Noguerón Martinez, Ana Fernández-Freire, Nieves del Pozo, Javier Cassinello, Martín Lázaro-Quintela, Fernando Arranz, Guillermo Crespo, María Teresa Abad, Mar Llorente, Alfredo Sánchez-Hernández, María José Juan

**Affiliations:** 1 Oncology Service, Complejo Hospitalario Universitario Ourense (CHOU), Ourense, Spain; 2 Oncology Service, Complejo Hospitalario Universitario A Coruña (CHUAC), A Coruña, Spain; 3 Oncology Service, Complejo Hospitalario Universitario de Santiago (CHUS), Santiago, Spain; 4 Oncology Service, Hospital Universitario Central de Asturias (HUCA), Oviedo, Spain; 5 Oncology Service, Hospital Universitario y Politécnico La Fe, Valencia, Spain; 6 Oncology Service, Hospital Ramón y Cajal, Madrid, Spain; 7 Oncology Service, Hospital Universitario de Salamanca, Salamanca, Spain; 8 Oncology Service, Hospital Virgen de la Arrixaca, Murcia, Spain; 9 Oncology Service, Hospital Clínico Universitario San Carlos, Madrid, Spain; 10 Oncology Service, Hospital de Sagunto, Valencia, Spain; 11 Oncology Service, Hospital Marina Baixa, Alicante, Spain; 12 Oncology Service, Complejo Hospitalario Universitario de Albacete (CHUA), Albacete, Spain; 13 Oncology Service, Hospital Universitario Torrecárdenas, Almería, Spain; 14 Oncology Service, Hospital Universitario del Vinalopó, Elche, Spain; 15 Oncology Service, Hospital Universitario de Guadalajara, Guadalajara, Spain; 16 Oncology Service, Complejo Hospitalario Universitario de Vigo, Vigo, Spain; 17 Oncology Service, Hospital Río Carrión, Palencia, Spain; 18 Oncology Service, Complejo Asistencial Universitario de Burgos, Burgos, Spain; 19 Oncology Service, Hospital Universitario de Basurto, Bilbao, Spain; 20 Oncology Service, Hospital General Universitario de Elda, Alicante, Spain; 21 Oncology Service, Consorcio Hospitalario Provincial de Castellón, Castellón, Spain; 22 Oncology Service, Fundación Instituto Valenciano de Oncología (IVO), Valencia, Spain

**Keywords:** Avelumab, Locally advanced/metastatic urothelial carcinoma, Overall survival, Progression-free survival, Toxicity

## Abstract

**Purpose:**

Maintenance avelumab has shown improved overall survival compared to chemotherapy alone in first-line treatment of advanced urothelial carcinoma. This study evaluates real-world evidence of avelumab as first-line maintenance therapy in patients with locally advanced or metastatic urothelial carcinoma (la/mUC).

**Methods/Patients:**

This was a multicenter, observational, retrospective and prospective study conducted in 22 Spanish centers. Patients were selected based on existing medical records of those treated with avelumab as first-line maintenance therapy before initiating the study (retrospective data), and those who continued to receive avelumab until the end of treatment or end of study (prospective data). Endpoints included median progression-free survival (mPFS), median overall survival (mOS) when available, PFS rate at 12 months (PFS12) and safety profile.

**Results:**

Of the 125 patients enrolled, 113 were evaluable. The median follow-up of avelumab treatment was 10.7 months. Disease progression was the main reason for discontinuation in 70 (61.9%) patients, with a median time to progression disease of 6.8 months. The survival probability was 21.4% for mPFS, with progression disease or death in 67.3% of patients; 44.9% for PFS12, with progression disease or death in 52.2% of patients; and 92.2% for mOS, with death in 2.6% of patients. Adverse events (AEs) were reported in 12.4% of patients; 65.0% of AEs not related to avelumab, and 35.0% were serious (SAEs).

**Conclusions:**

The real-world results support the effectiveness and manageable safety profile of avelumab as first-line in stage IV urothelial carcinoma. Further prospective studies with longer follow-up are warranted to confirm these findings.

## Introduction

Urothelial carcinoma (UC) is the most common type of bladder cancer (BC), located mostly in bladder (90%), but also in renal pelvis (8%), ureter or urethra (2%), according to the American Cancer Society and the National Comprehensive Cancer Network. UC is associated with significant morbidity and mortality; at diagnosis, approximately 30% of patients present with muscle-invasive BC, and 5% with metastatic disease [1]. BC incidence is relatively high, with 95,546 and 224,777 new cases diagnosed in the US and Europe, respectively, in 2022 [2]. In Spain, 22,295 new cases were anticipated by 2022, making BC the 6th leading cause of cancer-related deaths [2, 3]. Prognosis of UC remains poor, and there is a need for additional therapeutic options that may alleviate the burden on the healthcare system and improve the quality of life (QoL) of patients [4].

For several decades, the standard first-line treatment for locally advanced/metastatic Urothelial Carcinoma (la/mUC) was cisplatin in combination with gemcitabine (response rate of 60%), or carboplatin for cisplatin-ineligible patients (response rate of 50%). However, in both cases, progression occurs within 9 months, and median Overall Survival (OS) rarely exceeds 15 months [5–7].

UC exhibits high genomic instability, high Programmed Death-Ligand 1 (PD-L1) protein expression, and DNA damage-response mutations, with the ability to evade the immune system by downregulating tumor-antigen presentation, upregulating various immune checkpoints, and inactivating cytotoxic T cells [8]. By binding to PD-1 receptors present on T cells, PD-L1 delivers an inhibitory signal that suppresses T-cell activation and cytokine production, thereby allowing tumor cells to evade immune-mediated destruction. This mechanism has been associated with high-grade tumors and worse clinical outcomes [8].

The discovery of this immune evasion pathway has prompted interest in Immune Checkpoint Inhibitors (ICIs) targeting PD-1/PD-L1 as therapeutic agents in BC [8–11]. Clinical trials have demonstrated the antitumor activity of ICIs, including atezolizumab, nivolumab, pembrolizumab and avelumab, in patients with la/mUC, especially in those ineligible for platinum-based chemotherapy [12–15]. Although early response rates were modest (23–24%), these agents provided increased median OS with manageable adverse events (AEs), and were generally better tolerated than conventional chemotherapy [13, 15, 16].

After progression on a first-line chemotherapy, only 25%–55% of patients are eligible for second-line chemotherapy [17, 18], including pembrolizumab or atezolizumab, if no immunotherapy was previously administered. Second-line chemotherapy alternatives for patients not eligible for anti-PD-1/PD-L1 therapy include vinflunine and taxanes, with modest response rates (20%), and a median OS of less than 10 months [4, 17]. Notably, chemotherapy may prime the immune system by reducing immunosuppressive cells, supporting the rationale for immune-based maintenance strategies [10, 19].

Avelumab, a PD-L1 inhibitor, has been investigated as maintenance therapy for patients who respond or achieve stable disease after first-line chemotherapy. The phase Ib (JAVELIN Solid Tumour) [20] and the phase III (JAVELIN Bladder 100) trials [12], showed that avelumab plus best supportive care significantly improved median OS (21.4 months) and PFS (3.7 months). These results led to its approval by the FDA (June 2020) and EMA (January 2021), and its incorporation into both NCCN and ESMO guidelines as first-line maintenance therapy for la/mUC in SOC [18].

While randomized clinical trials (RCTs) remain the gold standard for new investigational drugs, real-world evidence (RWE) is increasingly important for validating trial findings and guiding regulatory decisions. Real-world data (RWD) can also identify unmet clinical needs, assess treatment duration, and support healthcare resource planning [21–26].

The present study aimed to evaluate the real-world evidence of avelumab as first-line maintenance therapy in patients with la/mUC previously treated with platinum-based chemotherapy.

## Patients and methods

### Study design and patients

This was a multicenter, observational, retrospective and prospective study performed at 22 Spanish hospitals, that expected to recruit 120 patients. Each participating center selected patients in chronological order, in accordance with the predefined selection criteria, and based on the information available in their medical records indicating that the patients had received or were still receiving avelumab as first-line maintenance therapy. Main inclusion criteria were adult (> 18 years of age) patients of both sexes diagnosed with la/mUC stage IV disease before first-line with carboplatin/cisplatin-based chemotherapy, with no disease progression after four-six cycles of chemotherapy according to the Response Evaluation Criteria in Solid Tumors (RECIST 1.1), and patients who started avelumab as maintenance therapy in first-line after 21/Jan/2021 and before 27/Apr/2022 (inclusive, from drug approval to before the national reimbursement price).

The study started in September 2022 and included two segments, a retrospective phase collecting information from all patients (deceased and alive) who received avelumab treatment before this date, and a prospective phase collecting information from the alive patients who continued to receive avelumab treatment until the End of Treatment/End of Study (EoT/EoS). For the prospective phase, patients were invited to participate in the study during a regular follow-up visit with the oncologist. Patient participation did not involve any change in treatment or care.

According to the Summary of Product Characteristics, the recommended dose of avelumab as monotherapy is 800 mg administered intravenously over 60 min every 2 weeks, followed by the recommended dosing schedule until disease progression or unacceptable toxicity. However, as this was an observational study in SOC, all patients treated with avelumab, regardless of dose and regimen, were included.

The study was registered in the Spanish Clinical Studies Register (REec), approved by the Research Ethics Committee for medicinal products (CEIm) at each center, and conducted in accordance with the principles of the Declaration of Helsinki and the International Conference on Harmonization (ICH) Good Clinical Practices, as well as local regulatory requirements.

Written informed consent was obtained from all patients except for deceased patients, in accordance with the EU Regulation 2016/679. Additionally, the electronic medical records of deceased patients were reviewed to ensure that they had not expressed in life opposition to the use of their data for investigational purposes.

### Study assessments

Descriptive information collected included demographic data; family history, medical comorbidities, ECOG performance status with smoking history, bone lesions and renal impairment; confirmation of diagnosis and staging, stratification, date of diagnosis, viral infections, and serology; molecular biomarkers PD-L1/PD1 and FGFR (if available); description of platinum-based first-line treatment including type of chemotherapy, number of cycles, response evaluation, treatment-free interval, etc.; and description of first-line avelumab maintenance, including dosage, duration and cycles, and response evaluation under standard clinical practice during the study.

The endpoints to evaluate effectiveness were: (1) median progression-free survival (mPFS) (primary endpoint), defined as the median time for patients from treatment initiation with avelumab to the date of progression event or death due to any cause; (2) progression-free survival at 12 months (PFS12) (secondary endpoint), defined as the percentage of alive patients and that remained progression free 12 months after treatment initiation with avelumab; (3) median overall survival (mOS) (secondary endpoint), defined as the median length of the time from the date patient initiates treatment with avelumab to the date of death.

Safety endpoints (secondary endpoints) were adverse events (AEs), serious AEs (SAEs) and treatment-related AEs (TRAEs) occurring during the prospective data collection period, and adverse drug reactions (ADRs) or serious ADRs (SADRs) occurring during the treatment period (retrospective and prospective). An AE/TRAE/ADR was considered serious if it resulted in death, was life-threatening, required hospitalization or prolongation of existing hospitalization, caused persistent or significant disability or incapacity, was a congenital anomaly or birth defect, or any other significant medical event. All these data were collected from the inclusion date until EoT/EoS.

### Statistical analysis

Categorical variables were presented as absolute and relative frequencies, and continuous variables as mean, median, standard deviation (SD), 95% confidence interval (95% IC), and range (minimum; maximum).

mPFS and mOS were estimated using the Kaplan–Meier method, with median and 95% CI and corresponding survival curve reported. For mPFS, patients without a real-world progression event or date of death were censored at the most recent visit with the treating oncologist or end of follow-up; for PFS12, patients without a real-world progression event or date of death were censored at month 12; and for mOS, patients not dead were censored at the most recent visit with the treating oncologist or end of follow-up.

The effectiveness and safety objectives were analyzed using a comprehensive sample of all eligible patients who met all selection criteria, with at least 14 days of follow-up data available for survival analysis.

All statistical analyses were performed using the SAS statistical package, version 9.4.

## Results

Of the 125 patients recruited between September 2022 and July 2023, 10 did not meet the selection criteria, and 2 were deceased patients from the Community of Madrid, where the local Ethics Committee—unlike other committees—only authorized the inclusion of living patients. These 12 patients were excluded from the analysis, resulting in a final evaluable cohort of 113 patients (74 alive and 39 deceased at baseline).

Baseline characteristics are presented in Table 1. The mean age of the patients was 69.6 years, 96 (85.0%) were male, the median time from diagnosis to initiation of avelumab was 6.8 months, and 90.9% of patients had invasive la/mUC.

**Table 1:** Baseline patient demographics and clinical characteristics

Parameter	Result
Patient demographics	
Age (years), mean ± SD	69.6 ± 8.5
Gender, n (%)	
Male	96 (85.0%)
Female	17 (15.0%)
Ethnic group, n (%)	
Caucasian	111 (98.2%)
Other	2 (1.8%)
Family and Medical history	
Family history of cancer, n (%)	
Yes	32 (28.3%)
No	81 (71.7%)
Smoking history^a^, n (%)	
Never smoked	16 (14.2%)
Smoker	28 (24.8%)
Never smoked	63 (55.8%)
Status and diagnosis	
ECOG performance status^a^, n (%)	
ECOG = 0	35 (31.3%)
ECOG = 1	71 (63.4%)
ECOG = 2	6 (5.4%)
Months since diagnosis of la/mUC to start of avelumab, median (range)	6.8 (2.1; 62.3)
Type of la/mUC^a^, n (%)	
Non-invasive	10 (9.1%)
Invasive	100 (90.9%)
Molecular biomarkers	
PD-L1 testing performed, n (%)	
Yes	32 (28.3%)
Positive	21 (65.6%)
Negative	11 (34.4%)
No	81 (71.7%)
PD-1 testing performed, n (%)	
Yes	0 (0.0%)
No	113 (100.0%)
FGFR testing performed, n (%)	
Yes	12 (10.6%)
Positive	1 (8.3%)
Negative	10 (83.3%)
Not evaluable	1 (8.3%)
No	101 (89.4%)

*ECOG* Eastern Cooperative Oncology Group, *FGFR* Fibroblast Growth Factor Receptor, *la/mUC* locally advanced/metastatic urothelial carcinoma

^a^Not reported in some cases

Treatment and response evaluation information is shown in Table 2. Regarding first-line platinum treatment, 54.0% of patients received cisplatin and 46.0% received carboplatin, for a median of 103 days and a median of 4.0 cycles administered. The first RECIST evaluation performed after completion of platinum treatment showed a complete response in 10.7%, a partial response in 63.4% and stable disease in 25.9% of patients.

**Table 2:** Treatment information

Parameter	Result
Platinum treatment received in first-line	
Type of platinum treatment, n (%)	
Cisplatin	61 (54.0%)
Carboplatin	52 (46.0%)
Median duration of cycles (days)^a^, median (range)	103 (44.0; 218.0)
Median number of cycles planned, median (range)	6.0 (3.0; 6.0)
Median number of cycles administered, median (range)	4.0 (4.0; 6.0)
RECIST evaluation (after platinum treatment was finished)^b^, n (%)	
Complete response	12 (10.7%)
Partial response	71 (63.4%)
Progressive disease^c^	0 (0.0%)
Stable disease	29 (25.9%)
Avelumab treatment	
Dose, n (%)	
800 mg	77 (68.1%)
Other dose	36 (31.9%)
Mean other dose (mg), mean ± SD	712.4 ± 124.4
Treatment ongoing, n (%)	
Yes	21 (18.6%)
No	92 (81.4%)
Median duration of cycles (days)^a,d^, median (range)	215.5 (1.0; 916.0)
Median number of cycles planned^2^, median (range)	16.5 (6.0; 24.0)
Median number of cycles administered, median (range)	
All patients	13.0 (1.0; 56.0)
Patients with treatment ongoing	35.0 (11.0; 52.0)
Patients with treatment ended	11.0 (1.0; 56.0)
RECIST evaluation (after avelumab treatment was finished)	
Retrospective period	
Response evaluated, n (%)	
Yes	109 (96.5%)
No	4 (3.5%)
Mean number of response evaluations by patient, mean ± SD	2.6 ± 1.6
Evaluation (from 298 response evaluations obtained), n (%)	
Complete response	37 (12.4%)
Partial response	49 (16.4%)
Progressive disease	76 (25.5%)
Stable disease	129 (43.3%)
Not evaluable	7 (2.3%)
Prospective period	
Response evaluated, n (%)	
Yes	29 (25.7%)
No	84 (74.3%)
Mean number of response evaluations by patient, mean ± SD	0.4 ± 0.8
Evaluation (from 48 response evaluations obtained), n (%)	
Complete response	7 (14.6%)
Partial response	6 (12.5%)
Progressive disease	8 (16.7%)
Stable disease	26 (54.2%)
Not evaluable	1 (2.1%)
Follow-up and End of study	
Median time in follow-up (months)^e^, median (range)	10.7 (1.4; 30.1)
Median time until progression disease (months)^e^, median (range)	6.8 (1.3; 26.5)
Treatment post avelumab	
Treatment^f^, n (%)	
Yes	54 (48.2%)
Chemotherapy	37 (68.5%)
Anti PD-1	0 (0.0%)
Anti PD-L1	0 (0.0%)
Antibody–drug conjugation	13 (24.1%)
Other	5 (9.3%)
No	58 (51.8%)
Median time since end of avelumab treatment (days)^g^, median (range)	35.5 (13.0; 384.0)
Ongoing, n (%)	
Yes	10 (18.5%)
No	44 (81.5%)
Median duration of treatment (days)^d,g^, median (range)	80.5 (1.0; 308.0)
RECIST evaluation (best response achieved with treatment post avelumab), n (%)	
Complete response	1 (1.9%)
Partial response	8 (14.8%)
Progressive disease	18 (33.3%)
Stable disease	15 (27.8%)
Not evaluable	12 (22.2%)

*RECIST* Response Evaluation Criteria in Solid Tumors

^a^Time elapsed between first and last cycle

^b^Not reported in some cases

^c^Progression disease should be 0 according to inclusion criteria

^d^Patients with treatment ended

^e^Time elapsed between start of avelumab treatment and end of study

^f^A single patient might receive more than one treatment

^g^Time elapsed between start and end of post treatment

Regarding avelumab treatment, 68.1% of patients received a dose of 800 mg; at the end of the follow-up, 21 (18.6%) patients were continuing treatment, with a median of 35.0 cycles administered, and 92 (81.4%) patients had discontinued treatment, with a median duration of cycles of 215.5 days and a median of 11.0 cycles administered. Almost all patients (96.5%) reported retrospective response evaluations to avelumab, showing complete response in 12.4%, partial response in 16.4%, progressive disease in 25.5%, and stable disease in 43.3% of patients. However, only 25.7% of patients reported prospective response evaluations, showing complete response in 14.6%, partial response in 12.5%, progressive disease in 16.7%, and stable disease in 54.2% of patients.

The median follow-up time (Table 2) from the start of avelumab treatment to the end of study was 10.7 months, being progression disease the main reason for discontinuation in 70 (61.9%) patients, and the median time until progression disease was 6.8 months.

Treatment post avelumab (Table 2) was reported by 54 (48.2%) patients, with a median of 35.5 days since end of avelumab treatment, and chemotherapy was the most frequent type of treatment in 68.5% of patients. Evaluation of the best response achieved showed complete response in 1.9%, partial response in 14.8%, progressive disease in 33.3%, and stable disease in 27.8% of patients.

**Fig. 1:**
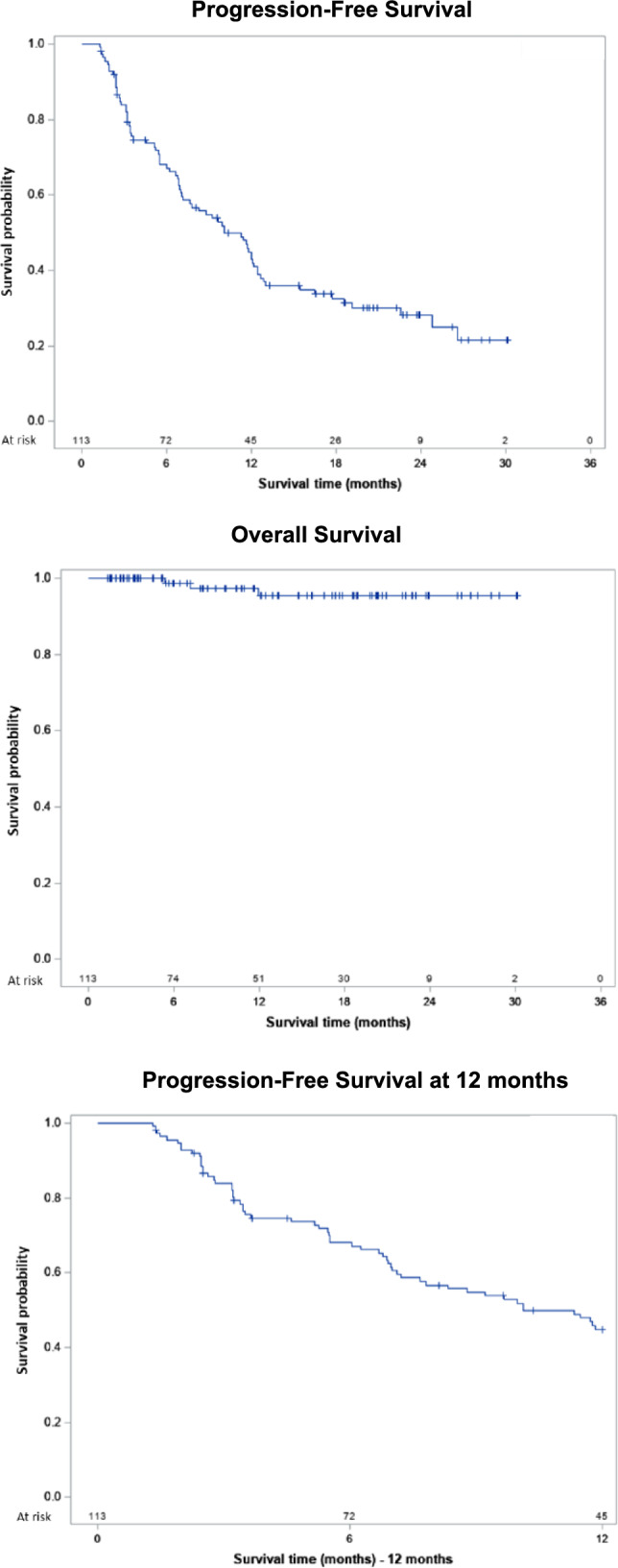
Survival curves. **a.** Progression-free survival. **b.** Overall survival. **c.** Progression-free survival at 12 months

The effectiveness analysis is shown in Table 3. For mPFS, considering that 76 (67.3%) patients reported a progression event or death from any cause, the survival probability was 0.2140 (21.4%) (Fig. 1A) with a median PFS of 10.1 months. For mOS, considering that only 3 (2.6%) patients were reported as dead during the 12 months of follow-up, the survival probability under avelumab was 0.9542 (95.4%) (Fig. 1B). For PFS12, considering that 59 (52.2%) patients reported a progression event or death from any cause, the survival probability was 0.4492 (44.9%) (Fig. 1C), with a median time until event or death of 10.1 months.

**Table 3:** Effectiveness analysis

Variable	Total n (%)	Event^a^ n (%)	Censored patients^b^ n (%)	Survival probability	Median time until event or death^c^ Median (95% IC)
PFS	113 (100.0%)	76 (67.3%)	37 (32.7%)	0.2140	10.1 (7.1–12.4)
OS	113 (100.0%)	3 (2.6%)	110 (97.4%)	0.9542	NC^d^
PFS12	113 (100.0%)	59 (52.2%)	54 (47.8%)	0.4492	10.1 (7.1–NC^e^)

*NC* not calculated, *OS* overall survival, *PFS* progression-free survival, *PFS12* progression-free survival at 12 months

^a^For PFS and PFS12: real-world progression event or death due to any cause, whichever occurs first; for OS: death due to any cause

^b^Censored patients at the end of the study

^c^For PFS and PFS12: months from avelumab first cycle until real-world progression event or death for any cause, whichever occurs first; for OS: months from avelumab first cycle until death due to any cause

^d^Not calculated because the survival probability was > 0.5

^e^Not calculated because the survival probability was near 0.5

Finally, the safety analysis is summarized in Table 4. Of the 113 participating patients, 14 (12.4%) reported a total of 20 adverse events (AEs), being 7 of them serious (SAEs). Of the 20 AEs, 13 (65.0%) were considered not related with study drug, while the other 7 (35.0%) were considered related with study drug (TRAEs). Of the 7 SAEs, 1 (14.03%) resulted in death, and 6 (85.7%) required hospitalization or prolongation of existing hospitalization. The SAE resulting in death, was a subcapsular hepatic hematoma, and was considered not related with the study drug. Of the 7 SAEs, 6 (85.7%) were considered not related with study drug and 1 (14.3%) was probably/likely related with study drug. Of the 7 TRAEs, 1 (14.3%) was serious, consisting in pancreatitis and requiring hospitalization or prolongation of existing hospitalization.

**Table 4:** Safety analysis

	Prospective period			Retrospective and prospective	
	AEs reported	SAEs reported	TRAEs reported	ADRs reported	SADRs reported
Patients with events reported n (%)					
Yes	14 (12.4%)	6 (5.3%)	6 (5.3%)	39 (34.5%)	4 (3.5%)
No	99 (87.6%)	107 (94.7%)	107 (94.7%)	74 (65.5%)	109 (96.5%)
*Total number of events reported*	*20*	*7*	*7*	*82*	*4*
Severity, n (%)					
Mild	10 (50.0%)	0 (0.0%)	4 (57.1%)	67 (81.7%)	0 (0.0%)
Moderate	4 (20.0%)	1 (14.3%)	2 (28.6%)	11 (13.4%)	0 (0.0%)
Severe	6 (30.0%)	6 (85.7%)	1 (14.3%)	4 (4.9%)	4 (100.0%)
Relation with study drug, n (%)					
Unlikely (non-related)	13 (65.0%)	6 (85.7%)	0 (0.0%)	–	–
Certain	1 (5.0%)	0 (0.0%)	1 (14.3%)	26 (31.7%)	2 (50.0%)
Probably/likely	4 (20.0%)	1 (14.3%)	4 (57.1%)	37 (45.1%)	2 (50.0%)
Possible	2 (10.0%)	0 (0.0%)	2 (28.6%)	18 (22.0%)	0 (0.0%)
Conditional/Unclassified	0 (0.0%)	0 (0.0%)	0 (0.0%)	0 (0.0%)	0 (0.0%)
Unassessable/Unclassifiable	0 (0.0%)	0 (0.0%)	0 (0.0%)	1 (1.2%)	0 (0.0%)
Action taken with study drug, n (%)					
No action taken	11 (55.0%)	2 (28.6%)	2 (28.6%)	65 (79.3%)	0 (0.0%)
Study drug held temporarily	6 (30.0%)	4 (57.1%)	3 (42.9%)	9 (11.0%)	2 (50.0%)
Dose reduced	0 (0.0%)	0 (0.0%)	0 (0.0%)	0 (0.0%)	0 (0.0%)
Study drug interrupted permanently	4 (20.0%)	2 (28.6%)	2 (28.6%)	8 (9.8%)	2 (50.0%)
Outcome, n (%)					
Recovered	10 (50.0%)	4 (57.1%)	3 (42.9%)	54 (65.9%)	2 (50.0%)
Recovering	4 (20.0%)	0 (0.0%)	3 (42.9%)	8 (9.8%)	0 (0.0%)
Recovered with sequelae	2 (10.0%)	2 (28.6%)	0 (0.0%)	4 (4.9%)	1 (25.0%)
Not recovered	3 (15.0%)	0 (0.0%)	1 (14.3%)	12 (14.6%)	1 (25.0%)
Fatal	1 (5.0%)	1 (14.3%)	0 (0.0%)	0 (0.0%)	0 (0.0%)
Unknown	0 (0.0%)	0 (0.0%)	0 (0.0%)	4 (4.9%)	0 (0.0%)
Ongoing, n (%)					
Yes	7 (35.0%)	0 (0.0%)	4 (57.1%)	23 (28.0%)	1 (25.0%)
No	13 (65.0%)	7 (100.0%)	3 (42.9%)	59 (72.0%)	3 (75.0%)
Median duration in days, median (range)	8.5 (1.0; 59.0)	8.0 (4.0; 12.0)	23.0 (4.0; 59.0)	29.5 (1.0; 316.0)	32.0 (4.0; 40.0)

*AE* adverse event, *ADR* adverse drug reaction, *SADR* serious adverse drug reaction, *SAE* serious adverse event, *TRAE* treatment related adverse event

Regarding the adverse drug reactions (ADRs) during the treatment period (including both retrospective and prospective periods), 39 (34.5%) of the 113 participating patients reported a total of 82 adverse drug reactions (ADRs), being 4 of them serious (SARDs). Of the 4 SARDs, 2 required hospitalization or prolongation of existing hospitalization (1 pancreatitis and 1 nephritis, both considered as probably related with the study drug) and 2 were other important medical events (1 hypertransaminasemia and 1 autoimmune hypothyroidism, both considered as certainly related with the study drug).

## Discussion

Untreated metastatic urothelial carcinoma of the bladder is associated with a median PFS between 3 and 6 months [17]. Combination platinum-based chemotherapy remains the SOC for first-line treatment of advanced urothelial carcinoma, although median PFS and OS typically do not exceed 9 and 15 months, respectively, with different combination regimens.

The primary objective of the present study was to evaluate the effectiveness of avelumab as first-line maintenance therapy in real-world clinical practice for patients with locally advanced or metastatic urothelial carcinoma, who had not progressed following four to six cycles of platinum-based chemotherapy.

The median PFS in this real-world study was 10.1 months, with a survival probability of 21.4%. These results suggest a potentially prolonged PFS with avelumab maintenance treatment compared to previously reported data. For instance, in the JAVELIN Bladder 100 trial, the median PFS was 3.7 months [12]. In a long-term follow-up study with avelumab, the median PFS was 5.5 months [27]. However, the median PFS is higher in real-life studies and similar to our findings, as shown in a German study where the median PFS was 6.2 months with a median follow-up of 8 months [28], and in a Portuguese cohort the median PFS was 9.8 months with a follow-up of 17.7 months [29].

In addition to the PFS data, it is noteworthy that the evolution of the RECIST evaluations during avelumab treatment showed an increase in the percentage of patients with stable disease over time, which may reflect disease control attributable to the effectiveness of avelumab. Post-avelumab treatment evaluation showed an expected increase in progressive disease, likely due to the natural course of the illness. In the present study, the median time to documented progression after initiating maintenance treatment was 6.8 months, providing further insight into treatment dynamics beyond standard survival metrics.

Regarding the differences observed in response evaluations between the retrospective and prospective segments of the study, it is important to clarify that the retrospective segment of the study includes a larger number of patients, as it encompasses data from both deceased and alive patients. This likely accounts for the higher percentage of disease progression observed in this group. In contrast, the prospective segment consists primarily of alive patients who were followed through to the end of the study, which may explain the higher proportion of stable disease reported.

Regarding OS, the survival analysis presented is limited by the small number of death events occurred under avelumab during the 12-month follow-up, which reduces statistical power and may affect the precision of the estimates. Nevertheless, these results reflect the real-world short-term outcomes in this population, where there were only 3 deaths during the 12 months of follow-up in this study.

This study has some limitations, such as the relatively short follow-up period, with a median duration of 10.7 months, which may be considered too short to evaluate survival parameters, and may restrict the ability to estimate longer-term outcomes with sufficient precision. Other studies with avelumab treatment found a mOS of 21.4 months with a median follow-up of more than 19 months, and a mOS of 23.8 months with a median follow-up of more than 38 months [12, 29].

There may be a potential selection bias in this study, as only patients who remained progression-free after platinum-based chemotherapy and initiated avelumab maintenance were included. This may have led to a sample with inherently better prognosis than the general urothelial carcinoma population. Additionally, treatment decisions in routine practice may be influenced by factors such as comorbidities, or clinician judgement, which were not systematically controlled in this study.

Another limitation may be the retrospective design, which introduces limitations in data completeness and consistency, leading to potential underreporting or misclassification.

Regarding safety, the profile of avelumab was consistent with previous reports, and no new safety signals were identified [12, 30]. Only 12.4% of patients in this study reported the presence of 20 AEs, and there was only a single SAE resulting in death, which was not attributed to avelumab. This findings may suggest a favorable safety profile; however it is important to acknowledge the inherent limitations in AE reporting in retrospective studies, which rely on routine documentation rather than active monitoring. For comparison, in the JAVELIN Bladder 100 trial, with a median treatment duration of 24.9 months, the incidence of AEs was 98.0% and 2 deaths were attributed to avelumab toxicity [12]. The lower incidence observed in the present study cohort reflects underreporting rather than a significantly better safety profile.

Despite these limitations, the present results contribute to the growing body of real-world evidence supporting the use of avelumab as maintenance therapy and highlight its potential benefit in a clinical setting. However, the data also emphasizes the need for prospective or registry-based studies with longer follow-up, standardized data collection, and broader patient inclusion criteria to confirm these findings. An extension of this study is currently under consideration to assess 24-month OS outcomes and address some of the limitations described above.

## Conclusions

The results of the present study are consistent with those of the phase III JAVELIN Bladder 100 trial and other real-world studies, supporting the effectiveness and manageable safety profile of avelumab in first-line in stage IV urothelial carcinoma. While these finding suggest that long-term avelumab treatment may be feasible and manageable in clinical practice, further prospective studies with longer follow-up are needed to confirm these observations.

## Data Availability

All relevant data are within the manuscript and its supporting information files. The data that support the findings of this study are available on request through the corresponding author and upon approval from the Spanish Oncology Genito Urinary Group (SOGUG).
